# Photobiomodulation for the Treatment of Primary Headache: Systematic Review of Randomized Clinical Trials

**DOI:** 10.3390/life12010098

**Published:** 2022-01-11

**Authors:** Andréa Oliver Gomes, Ana Luiza Cabrera Martimbianco, Aldo Brugnera Junior, Anna Carolina Ratto Tempestini Horliana, Tamiris da Silva, Elaine Marcílio Santos, Yara Dadalti Fragoso, Kristianne Porta Santos Fernandes, Samir Nammour, Sandra Kalil Bussadori

**Affiliations:** 1 Postgraduate Program in Rehabilitation Sciences, Universidade Nove de Julho (UNINOVE), Street Vergueiro, 235/249-Liberdade, São Paulo 05503-900, Brazil; an_oliver@hotmail.com (A.O.G.); tamiris.slv@hotmail.com (T.d.S.); 2 Postgraduate Program in Health and Environment, Universidade Metropolitana de Santos (UNIMES), São Paulo 05503-900, Brazil; analuizacabrera@hotmail.com (A.L.C.M.); elaine.marcilio@unimes.br (E.M.S.); yara.eegs@gmail.com (Y.D.F.); 3 Physics Institute of São Carlos (IFSC/USP), Universidade de São Paulo, São Paulo 05503-900, Brazil; aldo.brugnera@gmail.com; 4 Postgraduate Program in Biophotonics Applied to Health Sciences, Universidade Nove de Julho (UNINOVE), São Paulo 05503-900, Brazil; annacrth@gmail.com (A.C.R.T.H.); kristianneporta@gmail.com (K.P.S.F.); 5 Department of Dental Science, Faculty of Medicine, University of Liege, 4000 Liege, Belgium; s.namour@uliege.be

**Keywords:** pain, primary headache, photobiomodulation, low-level laser

## Abstract

The purpose of this study was to evaluate the efficacy and safety of photobiomodulation as an adjuvant treatment for primary headache. A systematic review of randomized clinical trials was performed. For such, electronic searches were performed in the MEDLINE, Embase, Cochrane Library, LILACS, PEDro, PsycInfo, Clinicaltrials.gov., and WHO/ICTRP databases, with no restrictions imposed regarding language or year of publication. We included studies that assessed any photobiomodulation therapy as an adjuvant treatment for primary headache compared to sham treatment, no treatment, or another intervention. The methodological assessment was conducted using the Cochrane Risk of Bias tool. The certainty of the evidence was classified using the GRADE approach. Four randomized clinical trials were included. Most of the included studies had an overall high risk of bias. Compared to sham treatment, photobiomodulation had a clinically important effect on pain in individuals with primary headache. Despite the benefits reported for other outcomes, the estimates were imprecise, and the certainty of the evidence was graded as low. These findings are considered insufficient to support the use of photobiomodulation in the treatment of primary headache. Randomized clinical trials, with higher methodological quality, are needed to enhance the reliability of the estimated effects.

## 1. Introduction

Headache is a common form of pain throughout the world. In most cases, the diagnosis of headaches is based entirely on patient history, as an isolated physical examination rarely provides adequate diagnostic information [1,2]. According to the World Health Organization, migraine is the second-highest leading cause of years lived with disability [3,4].

The International Headache Society published a classification system and operational diagnostic criteria for headache based on clinical consensus, classifying headache into primary (tension, migraine, or cluster) and secondary (e.g., caused by infection or vascular disease). The conventional treatment for primary headache is pharmacological (common analgesics or non-steroidal anti-inflammatory drugs). However, medication overuse can also be a cause of headache. In such cases, withdrawal should be counselled, so that the prophylactic treatment is effective [2].

Photobiomodulation with a low-level laser or light-emitting diode (LED) has been used for different therapeutic purposes since the end of the 1960s, when there was a considerable advance in the production of equipment and applications in the field of medicine [5,6,7]. Light at a wavelength in the red to infrared range of the spectrum (660 to 905 nm) is generally employed because such wavelengths can penetrate the skin and deeper tissues, resulting in a reduction in inflammation, pain relief, and accelerated regeneration of tissues, or act as an acupuncture needle [8,9].

Photobiomodulation is a recent adjuvant treatment for numerous neurological and psychological conditions. It seems to increase the metabolic capacity of neurons, and stimulate anti-inflammatory, anti-apoptotic, and antioxidant responses as neurogenesis and synaptogenesis [10]. The noninvasive delivery of light to the head and brain through photobiomodulation therapy is commonly called transcranial PBMT. With this method, light passes through a set of layers, such as the scalp, periosteum, bone, meninges, and dura mater, and partially reaches the brain’s cortical surface [10,11].

Therefore, this systematic review was performed to evaluate the efficacy and safety of photobiomodulation as an adjuvant treatment for primary headache.

## 2. Methods

This systematic review followed the methodological recommendations of the Cochrane Handbook for Systematic Reviews of Interventions [12] and the PRISMA statement [13] to ensure the quality of the report. In addition, the review protocol was prospectively registered with the International Prospective Register of Systematic Reviews (PROSPERO) under CRD42021223429.

### 2.1. Eligibility Criteria

#### 2.1.1. Types of Included Studies

We only considered randomized clinical trials (RCT) with parallel or crossover designs, independently of the publication status (complete article or abstract).

#### 2.1.2. Types of Participants

We included RCTs involving adults (18 years of age or older) diagnosed with primary headache.

#### 2.1.3. Types of Interventions

We included RCTs that assessed any photobiomodulation therapy as an adjuvant treatment for primary headache. The following comparators were considered: placebo (sham), no intervention, or different interventions, such as botulinum toxin type A, acupuncture, among others.

#### 2.1.4. Types of Outcome Measures

##### Primary Outcomes

- Pain intensity during a headache episode, measured by any validated scale or questionnaire, such as visual analogue scale (VAS), numeric rating scale, among others.
- Severity, duration, and frequency of episodes of headache.
- Serious adverse events during or after treatment that could lead to hospitalization or death.

##### Secondary Outcomes

- Minor adverse events, measured as the proportion of participants with at least one adverse event during or after treatment (e.g., exacerbation of pain, discomfort).
- Analgesics needed.
- Quality of life (measured by valid questionnaires, such as the SF-36).
- Patient acceptability.

The evaluation time of the outcomes was considered according to the follow-up analyzed in the included RCTs. In addition, the following intervals were considered for possible grouping in a meta-analysis: immediately after treatment, short term (up to three months after treatment), and long term (more than three months after treatment).

### 2.2. Search Strategies

Comprehensive and sensitive search strategies were conducted in the following six electronic databases on 14 February 2021, with no restrictions imposed regarding language or publication date: MEDLINE (via PubMed), EMBASE (via Elsevier), The Cochrane Central Register of Controlled Trials (CENTRAL) (via Wiley), Physiotherapy Evidence Database (PEDro), Literatura Latino Americana e do Caribeem Ciências da Saúde (LILACS (Latin American and Caribbean Health Sciences Literature) via Biblioteca Virtual emSaúde (Virtual Health Library)), and PsycInfo (via APA).

Ongoing clinical trials were searched in the following clinical trial registry databases: ClinicalTrials.gov (www.clinicaltrials.gov, accessed on 24 December 2021) and International Clinical Trials Registry Platform (ICTPR) of the World Health Organization (apps.who.int/trialsearch). In addition, the grey literature was searched in the OpenGrey database (www.opengrey.eu, accessed on 24 December 2021). The search strategies are presented in Supplementary Table S1. Finally, hand searches were also performed in the reference lists of relevant studies.

### 2.3. Study Selection and Data Extraction

The references retrieved by the search strategies were selected by two authors, independently, through the Rayyan platform [14]. After removing the duplications, the authors analyzed the titles and abstracts, and studies that did not meet the eligibility criteria were excluded. In the second stage, studies with potential inclusion were analyzed in full text to decide whether to include or exclude. A third reviewer solved disagreements.

Two authors independently extracted the following information on included RCTs using a form previously prepared in Microsoft Office Excel^®^: sample size, characteristics of the participants (age, sex, and duration of pain), aspects of the intervention and comparator groups, analyzed outcomes, follow-up time and results. When necessary, the authors of the trials were contacted to provide additional information or test data.

### 2.4. Methodological Quality Assessment of the Included Studies (Risk of Bias)

The methodological quality of the included studies was assessed by two independent reviewers using the Cochrane Risk of Bias (RoB) table [12]. This tool comprises the following seven domains: random sequence generation, allocation concealment, blinding of participants and personnel, blinding of outcome assessors, incomplete outcome data, selective reporting and other sources of bias. Each included RCT was classified as having a low, unclear, or high risk of bias for each domain.

### 2.5. Data Synthesis

When possible (homogeneous studies and available data), meta-analyses were planned to be performed using the Review Manager 5.4.1 software (RevMan 5.4.1) with a random effects model. The measure of effect used for data on dichotomous outcomes was the relative risk (RR), and for continuous outcomes, the difference in means (DM), both with a 95% confidence interval (95% CI). The heterogeneity between included studies was planned to be assessed by the I^2^ statistic, where greater than 50% is considered substantial heterogeneity [12]. However, in this systematic review, it was not possible to group data from included studies into a meta-analysis with considerable clinical variability between them. We also planned to conduct a publication bias analysis if more than ten studies were included in the meta-analysis.

### 2.6. Certainty of the Evidence

The certainty of the body of evidence was appraised for each outcome using the Grading of Recommendations Assessment, Development, and Evaluation (GRADE) approach [15].

## 3. Results

### 3.1. Search Results

**Figure 1:**
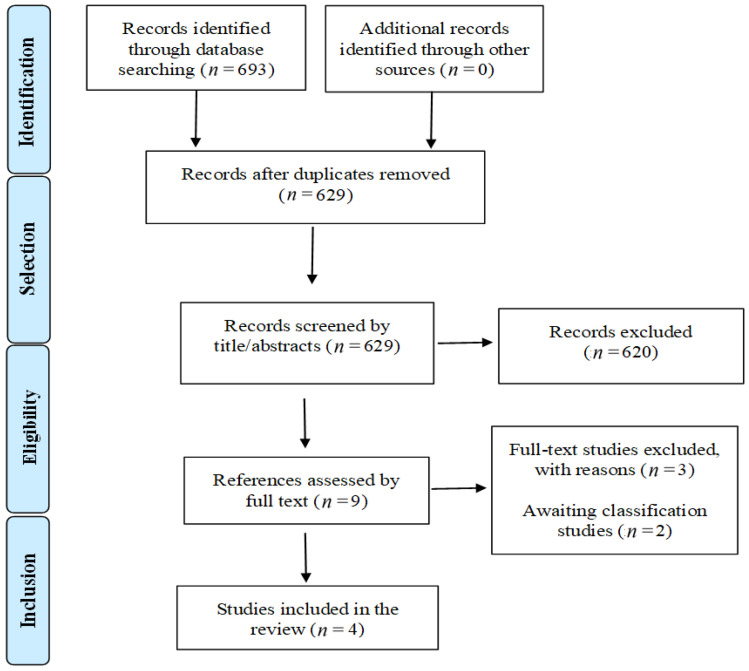
PRISMA flow of the study selection process.

The search strategies retrieved 693 records. After removing 64 duplicates, 629 records were analyzed based on the title and abstract, and 620 were excluded for not meeting the inclusion criteria. Nine studies were analyzed by full text, and the following three were excluded: one for not performing the comparison of interest [16], one for not being an RCT, [17], and one for combining therapeutic interventions [18]. Two ongoing studies were identified (CTRI/2020/05/024968 and NCT02969642). After the entire selection process, four RCTs [19,20,21,22] were included in this systematic review. Figure 1 details the article selection process.

### 3.2. Characteristics of Studies Included in the Review

Table 1 displays the characteristics of the included RCTs. The studies were published between 1997 and 2020. One was published in Italy [19], one in Iran [20], one in Brazil [21], and one did not report the origin of the study [22]. The four RCTs involved 174 participants and evaluated photobiomodulation therapy to treat primary headache. The main inclusion criterion was the frequency of headache, with more than 15 days of pain per month. The main exclusion criteria were the overuse of analgesics and pre-existing conditions as other causes of headache.

All the included studies avoided the use of concomitant analgesics and prophylactic medication.

### 3.3. Methodological Quality Assessment of the Included Studies (Risk of Bias)

**Figure 2:**
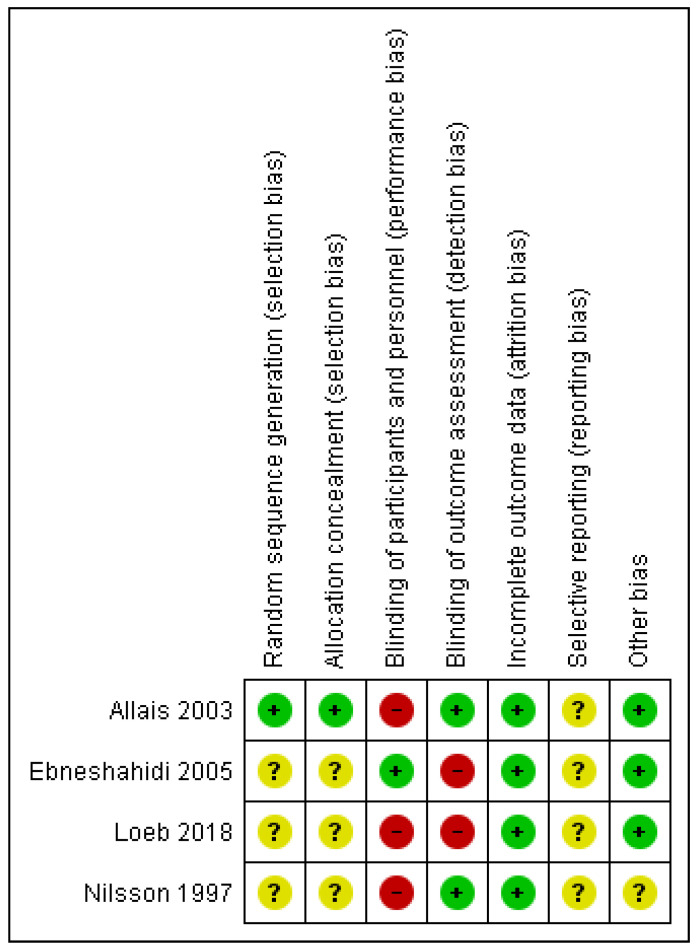
Risk of Bias assessment for each included study. (+) = low risk of bias; (?) = unclear risk of bias; (-) = high risk of bias.

Figure 2 summarizes the methodological quality assessment. Three of the four studies did not describe the randomization process or allocation concealment, and were classified as having an uncertain risk of bias for these domains. Three studies [19,21,22] were judged to have a high risk of bias for blinding of the participants and therapists, due to the failure to provide sufficient information; as the interventions were different and the outcomes analyzed were subjective, it is likely that blinding was not performed, which could have influenced the final results. Similarly, two studies [20,21] were judged to have a high risk of bias regarding the blinding of the assessors of the outcomes. None of the four studies reported having registered the protocol of the study. One study [22] was judged to have an uncertain risk of bias regarding “other sources of bias” due to the failure to describe the initial characteristics of the participants to enable a comparison of the groups at baseline.

### 3.4. Effects of Intervention

#### 3.4.1. Comparison 1. Low-Level Laser Therapy (LLLT) versus Sham

A study with 50 participants [20] compared LLLT to sham (10 sessions over three weeks), and assessed the following outcomes after 2 months of treatment:

- Improvement in pain: Pain was assessed using the visual analogue scale (VAS). An improvement in pain was found favouring the LLLT group (mean −2.0 ± 6.3 versus 0 ± 0; *p* < 0.001).
- Frequency of headache episodes per day: an important reduction in headache episodes was found in the LLLT group compared to the sham group (−8.0 ± 21.5 versus 0.0 ± 0; *p* < 0.001).
- Duration of episodes (in hours): an improvement was found favoring the LLLT group (−4.0 ± 7.5 versus 0.0 ± 0.0; *p* < 0.001).

#### 3.4.2. Comparison 2: LLLT versus Botulinum Toxin Type A (BTX-A)

One study [21] compared the effects of LLLT to BTX-A, evaluating the following outcomes after 3 months of treatment:

- Frequency of headache episodes: no significant difference between groups was found regarding the reduction in episodes (*p* = 0.22).
- Adverse events: some patients in the LLLT group described a burning sensation at the application points, which disappeared at the end of the application.
- Use of analgesics: no significant difference between groups was found at the end of treatment (*p* = 0.12).

#### 3.4.3. Comparison 3. LLLT versus Acupuncture

A study with 60 participants [9] compared the effect of infrared LLLT (ten 30 min sessions) to acupuncture (twice per week during the first week and weekly during the subsequent six weeks). The effect estimates showed a change in direction throughout the evaluation period of the study, as follows:

- Immediately after treatment: no significant difference in the frequency of episodes of headache (days/month) (mean difference (MD): 0.38; 95% confidence interval [CI]: −2.43 to 3.19; *p* = 0.79).
- One month after treatment: a significant reduction in the frequency of episodes was found in the LLLT group compared to the acupuncture group (MD −6.79; 95% CI: −9.79 to −3.79; *p* < 0.00001).
- Four months after treatment: an increase in the number of episodes was found in the LLLT group, and a significant reduction was found in the acupuncture group (MD: 5.39; 95% CI: 2.96 to 7.82; *p* < 0.00001).

The authors reported no adverse events stemming from the interventions during the study period.

#### 3.4.4. Comparison 4. LLLT versus Transcutaneous Electrical Nerve Stimulation (TENS)

A study with 40 participants [19] compared the effect of infrared LLLT (ten 25 min sessions on alternate days) to TENS (ten 30 min daily sessions). No significant difference between groups was found regarding the reduction in the frequency of episodes of headache (days/month) at any of the evaluation times, as follows:

- Immediately after treatment: (MD: −0.01; 95% CI: −2.97 to 2.95; *p* = 0.99);
- One month after treatment (short term) (MD: 0.18; 95% CI: −3.43 to 3.79; *p* = 0.92);
- Four months after treatment (long term) (MD: −0.89; 95% CI: −3.30 to 1.52; *p* = 0.47).

The authors reported no adverse events stemming from the interventions during the study period.

None of the included studies assessed the quality of life and patient acceptability.

### 3.5. Certainty of the Evidence

The certainty of the body of evidence was classified as very low for all the outcomes analyzed in all the comparisons, due to the methodological limitations, small sample sizes, and imprecision. This indicated that we are uncertain about the effects of photobiomodulation for treating primary headache.

## 4. Discussion

This systematic review assessed the effects of photobiomodulation as adjuvant therapy for primary headache. This intervention has been extensively investigated for several clinical conditions, given its possible effects on pain reduction, muscle relaxation, and tissue regeneration, consequently to metabolic, anti-inflammatory, and/or antioxidant responses [10,11]. The results of this systematic review showed no relevant difference between PBM therapy and the following interventions evaluated in the included studies: analgesic therapy using TENS, acupuncture, and botulinum toxin type A [19]. Some studies [5,23,24] found that photobiomodulation was not superior to other therapies for the treatment of headaches, whereas another study [20] suggested improvements in pain and the frequency and duration of episodes of headache compared to sham treatment. In addition, no adverse events were reported, indicating this intervention’s safety. Nevertheless, these findings should be interpreted with caution, given the low methodological quality of the available evidence until now. Other studies [15,19,22], with the same level of evidence, suggested that photobiomodulation was not superior to other manual therapies, such as cervical manipulation and deep friction massage. However, the use of analgesic medication was not reported, impeding an assessment of the real effect of these treatments. It is worth mentioning that none of the included studies assessed clinically relevant outcomes, such as quality of life and patient satisfaction. These outcomes could directly assess the patient report, regarding treatment and its impact on the routine.

Four RCTs [19,20,21,22] were included in the present systematic review, most of which had a high risk of bias. The certainty of the body of evidence was classified as very low, which indicates that there are uncertainties about the effects of photobiomodulation for primary headache, and more significant randomized clinical trials are required. Blinding of the participants, allocation concealment, and intention-to-treat analysis were rarely used. Moreover, flaws were detected that downgraded the certainty of the evidence and reduced the confidence in the results. The majority of the RCTs included in this review had a small sample size (average of 18 participants per group) and either failed to describe the calculation of the sample size or described it inadequately, likely due to the considerable heterogeneity in the photobiomodulation parameters (irradiance, quantity of energy delivered, and duration and frequency of the treatments), characteristics of the participants, selection of outcome measures, and methodological quality.

The choice of dosimetric parameters is crucial to the effectiveness of photobiomodulation. Energy quantities inside the recommended therapeutic window were recommended for many diseases [25]. Unfortunately, we have no ideal parameters for primary headache. Thus, the interpretation of the results requires an assessment of the adequacy of the dosimetric parameters for photobiomodulation [5].

Moreover, most trials in the present review reported these parameters inadequately or insufficiently. Thus, information on the dosimetric parameters provided in all the RCTs [19,20,21,22] was incomplete, which could lead to difficulty in reproducing the protocol and underscores the need to exercise caution when interpreting the results and conclusions of the present systematic review.

Considerable dosimetric variation was found among the four clinical trials; the total energy ranged from 6 to 12 J per point, the irradiation time ranged from 33 to 60 s, and the average output power ranged from 27 to 100 mW. Furthermore, these parameters were not described in all the studies. The studies also failed to report the source of the choice of dosimetric parameters (the study used as a guide), and the optimization of these parameters was not discussed.

One study [20], with 50 participants, evaluated laserpuncture compared to sham, regarding pain and the frequency and duration of episodes of pain, reporting improvements in all these outcomes.

In comparing the effects of LLLT and BTX-A on the frequency of episodes of headache, [21] reported that treatments had the same efficacy with regards to the “use of analgesic” outcome.

Another study [19] compared the effect of infrared LLLT to acupuncture. The effect estimates showed conflicting changes depending on the time point. No significant difference between groups was found immediately after treatment. In contrast, the laser group exhibited better results than the acupuncture group one month after treatment, and the acupuncture group exhibited better results than the laser group four months after treatment. Although acupuncture studies on primary headache are starting to be realized [23], there is no evidence of the effect of acupuncture on migraine without aura [24]. In addition, electroacupuncture is one of several effective treatments for migraine pain. However, they conclude that multicentre studies with large sample sizes and well-designed randomized controlled trials are needed [26].

In comparing infrared LLLT and TENS [19], no significant difference between the groups was found, regarding the frequency of episodes of headache at any of the evaluation times.

Besides the inherent differences of each study, regarding the population, dosimetric parameters, and groups studied, divergences were found regarding the behaviour of the treatments over time. Moreover, the results should be interpreted with caution, due to the low quality of evidence.

One study [19] reported an absence of adverse effects with both treatments (LLLT and acupuncture). Another study [24] reported that some patients in the LLLT group described a burning sensation at the application points, which disappeared at the end of the application, and another study failed to evaluate adverse effects [18].

The strong points of the present review are the use of a sensitive search strategy in six databases and hand searches of manuscript reference lists, with no restrictions imposed regarding the language or date of publication. Moreover, two independent reviewers screened the trials, data extraction, and appraisal of methodological quality, as recommended by the Cochrane Handbook for Systematic Reviews of Intervention, and the certainty of the evidence was carefully appraised using the GRADE approach [15].

Future studies need to be based on the Consolidated Standards of Reporting Trials (CONSORT statement) [27]. Moreover, all photobiomodulation variables should be described clearly and thoroughly, preferably in a table to facilitate the visualization of the dosimetric parameters. Finally, randomized clinical trials with higher methodological quality, an increased number of participants, and long-term follow-up are needed to support photobiomodulation as adjuvant therapy for primary headache.

## 5. Conclusions

The current evidence does not support the use of photobiomodulation to reduce pain and the frequency of episodes of primary headache. Further randomized clinical trials, with adequate sample sizes and methodological quality, are needed to investigate the efficacy and safety of photobiomodulation as an adjuvant treatment for primary headache.

## Figures and Tables

**Table 1:** Main characteristics of the included studies.

Author/Year and Country	Population	Intervention	Comparators	Outcome Measure and Follow-Up	Main Results	Funding Sources
Allais et al., 2003, Italy [19]	*n* = 60 Age 21.6 years 100% women Headache more than 15 days/months, in previous six months	Infra LLLT 904 nm (*n* = 20) Intensity: 27 mV Time: 60 s/point Mode: NR 10 sessions, 25 min each Acupuncture points: GB20, GV20, GB14, Ex-HN5	10 sessions of acupuncture (*n* = 20) 10 sessions of TENS (*n* = 20) Points: GB20, GV20, GB14, Ex-HN5	Number of headache episodes (days/month) Assessed at 1 and 2 months	Compared to acupuncture, LLLT may result in frequency of headache episodes improvement after the following: 1 month (MD −6.79; 95% CI −9.79 to −3.79) 4 months (MD: 5.39; 95% CI 2.96 to 7.82) Compared to TENS, no differences were observed.	NR
Ebneshahidi et al., 2005, Iran [20]	*n* = 50 Age: 25–54 years 90% women Headache more than 15 days/months	LLLT 830 nm (*n* = 25) low energy laser radiation Intensity: 39 mV/cm^2^ Time: 43 s/point Continuous mode Dose: 1.3 J 10 sessions Acupuncture points: GB14, GB20, L14, and LU7	10 sessions of sham (*n* = 25) Energy output: zero Acupuncture points: GB14, GB20, L14, and LU7	Pain intensity (VAS) Duration of episodes (hours) Number of headache episodes (days/month) Assessed every 1 month, for 3 months	LLLT may result in a marginal improvement in the following: pain (−2.0 versus 0.0 VAS points) frequency of headache episodes (−8.0 versus 0.0 days) duration of headache episodes (−4.0 versus 0.0 h)	NR
Loeb et al., 2018, Brazil [21]	*n* = 36 Age: 20–65 years 90% women Headache more than 15 days/months	LLLT 808 nm (*n* = 18) Intensity: 100 mV Time: 33 s/point Continuous mode Dose: 120 J/cm^2^ 10 sessions, twice a week Cranial points	BTX-A (*n* = 18) Same cranial points	Number of headache episodes (days/month) Adverse events Use of medication Assessed every 1 month, for 3 months	LLLT may result in a non-significant difference in the following: frequency of headache episodes (*p* = 0.22) use of medication (*p* = 0.12)	CNPq, FAPESP, CAPES, Instituto Nacional de Neurociência Translacional (INNT)
Nilsson et al., 1997, Country NR [22]	*n* = 53 Age NR Sex NR Frequency NR	6 sessions of LLLT (type and parameters NR) Trigger points in the upper cervical region	Cervical manipulation Deep friction massage	Number of headache episodes (days) Assessed at 5 weeks	LLLT may result in a decreased number of headache hours per day (*p* = 0.03)	NR

CI: confidence interval; BTX-A: botulinum toxin type A; J: Joules; LLLT: low-level laser therapy; MD: mean difference; *n*: number of patients; NR: not reported; s: seconds; TENS: transcutaneous electrical nerve stimulation; VAS: visual analogue.

## Data Availability

Not applicable.

## Supplementary Materials

The following supporting information can be downloaded at: https://www.mdpi.com/article/10.3390/life12010098/s1, Table S1: Search strategies for each database.
