# Supplementary material for: Photobiomodulation for the Treatment of Primary Headache: Systematic Review of Randomized Clinical Trials
Source: Life (Basel). 2022 Jan 11;12(1):98. doi: 10.3390/life12010098 (PMC8781567; doi:10.3390/life12010098)
Supplement: Supplementary file 1 [file life-12-00098-s001.zip › life-1501490-supplementary.pdf]

Supplementary Table S1. Search strategies for each database.

**MEDLINE (via PubMed)**	#1 "Headache"[Mesh] OR Headaches OR (Head Pain) OR (Head Pains) OR (Pain, Head) OR (Pains, Head) OR Cephalodynia OR Cephalodynias OR (Cranial Pain) OR (Cranial Pains) OR (Pain, Cranial) OR (Pains, Cranial) OR Cephalalgia OR Cephalalgias OR Cephalgia OR Cephalgias OR (Generalized Headache) OR (Generalized Headaches) OR (Headache, Generalized) OR (Headaches, Generalized) OR (Ocular Headache) OR (Headache, Ocular) OR (Headaches, Ocular) OR (Ocular Headaches) OR (Orthostatic Headache) OR (Headache, Orthostatic) OR (Headaches, Orthostatic) OR (Orthostatic Headaches) OR (Vertex Headache) OR (Headache, Vertex) OR (Headaches, Vertex) OR (Vertex Headaches) OR (Retro-Ocular Headache) OR (Headache, Retro-Ocular) OR (Headaches, Retro-Ocular) OR (Retro Ocular Headache) OR (Retro-Ocular Headaches) OR (Sharp Headache) OR (Headache, Sharp) OR (Headaches, Sharp) OR (Sharp Headaches) OR (Throbbing Headache) OR (Headache, Throbbing) OR (Headaches, Throbbing) OR (Throbbing Headaches) OR (Unilateral Headache) OR (Headache, Unilateral) OR (Headaches, Unilateral) OR (Unilateral Headaches) OR Hemicrania OR (Bilateral Headache) OR (Bilateral Headaches) OR (Headache, Bilateral) OR (Headaches, Bilateral) OR (Periorbital Headache) OR (Headache, Periorbital) OR (Headaches, Periorbital) OR (Periorbital Headaches)<br>#2 "Headache Disorders, Primary"[Mesh] OR (Primary Headache Disorders)OR (Disorder, Primary Headache)OR (Disorders, Primary Headache)OR (Headache Disorder, Primary)OR (Primary Headache Disorder)OR (Primary Cough Headache)OR (Headache, Primary Cough) OR (Cough Headache)OR (Headache, Cough) OR (Benign Cough Headache) OR (Benign Cough Headaches)OR (Cough Headache, Benign)OR (Cough Headaches, Benign)OR (Headache, Benign Cough)OR (Headaches, Benign Cough)OR (Primary Thunderclap Headache) OR (Headache, Primary Thunderclap)OR (Thunderclap Headache)OR (Headache, Thunderclap)OR (Primary Exertional Headache)OR (Headache, Primary Exertional) OR (Benign Exertional Headache)OR (Benign Exertional Headaches)OR (Exertional Headache, Benign)OR (Exertional Headaches, Benign)OR (Headache, Benign Exertional)OR (Headaches, Benign Exertional)OR (Primary Stabbing Headache)OR (Headache, Primary Stabbing)OR (Ice-Pick Headache)OR (Headache, Ice-Pick)OR (Headaches, Ice-Pick)OR (Ice Pick Headache)OR(Ice-Pick Headaches)OR(Stabbing Headache)OR(Headache, Stabbing)OR(Hypnic Headache)OR(Headache,

	Hypnic)OR(Hypnic Headache Syndrome)OR(Headache Syndrome, Hypnic)OR(Headache Syndromes, Hypnic)OR(Hypnic Headache Syndromes)OR(Alarm Clock Headache)OR(Alarm Clock Headaches)OR(Clock Headache, Alarm)OR(Clock Headaches, Alarm)OR(Headache, Alarm Clock)OR(Headaches, Alarm Clock)<br>#3 #1 OR #2<br>#4 "Low-Level Light Therapy" [Mesh]OR (Light Therapies, Low-Level) OR (Light Therapy, Low-Level) OR (Low Level Light Therapy) OR (Low-Level Light Therapies) OR (Therapies, Low-Level Light) OR (Therapy, Low-Level Light) OR (Photobiomodulation Therapy) OR (Photobiomodulation Therapies) OR (Therapies, Photobiomodulation) OR (Therapy, Photobiomodulation) OR (LLLT) OR (Laser Therapy, Low-Level) OR (Laser Therapies, Low-Level) OR (Laser Therapy, Low Level) OR (Low-Level Laser Therapies) OR (Laser Irradiation, Low-Power) OR (Irradiation, Low-Power Laser) OR (Laser Irradiation, Low Power) OR (Low-Power Laser Therapy) OR (Low Power Laser Therapy) OR (Laser Therapy, Low-Power) OR (Laser Therapies, Low-Power) OR (Laser Therapy, Low Power) OR (Low-Power Laser Therapies) OR (Low-Level Laser Therapy) OR (Low Level Laser Therapy) OR (Low-Power Laser Irradiation) OR (Low Power Laser Irradiation) OR (Laser Biostimulation) OR (Laser Phototherapy) OR (Phototherapy, Laser)<br>#5 #3 AND #4(128)
**EMBASE**<br>**(via Elsevier)**	# 1 'headache'/exp OR Headaches OR 'Head Pain' OR 'Head Pains' OR 'Pain, Head' OR 'Pains, Head' OR Cephalodynia OR Cephalodynias OR 'Cranial Pain' OR 'Cranial Pains' OR 'Pain, Cranial' OR 'Pains, Cranial' OR Cephalalgia OR Cephalalgias OR Cephalgia OR Cephalgias OR 'Generalized Headache' OR 'Generalized Headaches' OR 'Headache, Generalized' OR 'Headaches, Generalized' OR 'Ocular Headache' OR 'Headache, Ocular' OR 'Headaches, Ocular' OR 'Ocular Headaches' OR 'Orthostatic Headache' OR 'Headache, Orthostatic' OR 'Headaches, Orthostatic' OR 'Orthostatic Headaches' OR 'Vertex Headache' OR 'Headache, Vertex' OR 'Headaches, Vertex' OR 'Vertex Headaches' OR 'Retro-Ocular Headache' OR 'Headache, Retro-Ocular' OR 'Headaches, Retro-Ocular' OR 'Retro Ocular Headache' OR 'Retro-Ocular Headaches' OR 'Sharp Headache' OR 'Headache, Sharp' OR 'Headaches, Sharp' OR 'Sharp Headaches' OR 'Throbbing Headache' OR 'Headache, Throbbing' OR 'Headaches, Throbbing' OR 'Throbbing Headaches' OR 'Unilateral Headache' OR 'Headache, Unilateral' OR 'Headaches, Unilateral' OR 'Unilateral Headaches' OR

	Hemicrania OR 'Bilateral Headache' OR 'Bilateral Headaches' OR 'Headache, Bilateral' OR 'Headaches, Bilateral' OR 'Periorbital Headache' OR 'Headache, Periorbital' OR 'Headaches, Periorbital' OR 'Periorbital Headaches'<br>#2 'photobiomodulation'/exp OR 'photobiomodulation therapy'/exp OR 'low level laser therapy'/exp OR 'Light Therapies, Low-Level' OR 'Light Therapy, Low-Level' OR 'Low Level Light Therapy' OR 'Low-Level Light Therapies' OR 'Therapies, Low-Level Light' OR 'Therapy, Low-Level Light' OR 'Photobiomodulation Therapy' OR 'Photobiomodulation Therapies' OR 'Therapies, Photobiomodulation' OR 'Therapy, Photobiomodulation' OR 'LLLT' OR 'Laser Therapy, Low-Level' OR 'Laser Therapies, Low-Level' OR 'Laser Therapy, Low Level' OR 'Low-Level Laser Therapies' OR 'Laser Irradiation, Low-Power' OR 'Irradiation, Low-Power Laser' OR 'Laser Irradiation, Low Power' OR 'Low-Power Laser Therapy' OR 'Low Power Laser Therapy' OR 'Laser Therapy, Low-Power' OR 'Laser Therapies, Low-Power' OR 'Laser Therapy, Low Power' OR 'Low-Power Laser Therapies' OR 'Low-Level Laser Therapy' OR 'Low Level Laser Therapy' OR 'Low-Power Laser Irradiation' OR 'Low Power Laser Irradiation' OR 'Laser Biostimulation' OR 'Laser Phototherapy' OR 'Phototherapy, Laser'<br>#3#1AND #2<br>#4 AND [embase]/lim NOT ([embase]/lim AND [medline]/lim) (80)
**The Cochrane Central Register of Controlled Trials (CENTRAL) (via Wiley)**	#1 MeSH descriptor: [Headache] explode all trees<br>#2 Headaches OR (Head Pain) OR (Head Pains) OR (Pain, Head) OR (Pains, Head) OR Cephalodynia OR Cephalodynias OR (Cranial Pain) OR (Cranial Pains) OR (Pain, Cranial) OR (Pains, Cranial) OR Cephalalgia OR Cephalalgias OR Cephalgia OR Cephalgias OR (Generalized Headache) OR (Generalized Headaches) OR (Headache, Generalized) OR (Headaches, Generalized) OR (Ocular Headache) OR (Headache, Ocular) OR (Headaches, Ocular) OR (Ocular Headaches) OR (Orthostatic Headache) OR (Headache, Orthostatic) OR (Headaches, Orthostatic) OR (Orthostatic Headaches) OR (Vertex Headache) OR (Headache, Vertex) OR (Headaches, Vertex) OR (Vertex Headaches) OR (Retro-Ocular Headache) OR (Headache, Retro-Ocular) OR (Headaches, Retro-Ocular) OR (Retro Ocular Headache) OR (Retro-Ocular Headaches) OR (Sharp Headache) OR (Headache, Sharp) OR (Headaches, Sharp) OR (Sharp Headaches) OR (Throbbing Headache) OR (Headache, Throbbing) OR (Headaches, Throbbing) OR (Throbbing Headaches) OR (Unilateral Headache) OR (Headache, Unilateral) OR (Headaches, Unilateral) OR

	(Unilateral Headaches) OR Hemicrania OR (Bilateral Headache) OR (Bilateral Headaches) OR (Headache, Bilateral) OR (Headaches, Bilateral) OR (Periorbital Headache) OR (Headache, Periorbital) OR (Headaches, Periorbital) OR (Periorbital Headaches)<br>#3 MeSH descriptor: [Headache Disorders, Primary] explode all trees<br>#4 (Primary Headache Disorders) OR (Disorder, Primary Headache) OR (Disorders, Primary Headache) OR (Headache Disorder, Primary) OR (Primary Headache Disorder) OR (Primary Cough Headache) OR (Headache, Primary Cough) OR (Cough Headache) OR (Headache, Cough) OR (Benign Cough Headache) OR (Benign Cough Headaches) OR (Cough Headache, Benign) OR (Cough Headaches, Benign) OR (Headache, Benign Cough) OR (Headaches, Benign Cough) OR (Primary Thunderclap Headache) OR (Headache, Primary Thunderclap) OR (Thunderclap Headache) OR (Headache, Thunderclap) OR (Primary Exertional Headache) OR (Headache, Primary Exertional) OR (Benign Exertional Headache) OR (Benign Exertional Headaches) OR (Exertional Headache, Benign) OR (Exertional Headaches, Benign) OR (Headache, Benign Exertional) OR (Headaches, Benign Exertional) OR (Primary Stabbing Headache) OR (Headache, Primary Stabbing) OR (Ice-Pick Headache) OR (Headache, Ice-Pick) OR (Headaches, Ice-Pick) OR (Ice Pick Headache) OR (Ice-Pick Headaches) OR (Stabbing Headache) OR (Headache, Stabbing) OR (Hypnic Headache) OR (Headache, Hypnic) OR (Hypnic Headache Syndrome) OR (Headache Syndrome, Hypnic) OR (Headache Syndromes, Hypnic) OR (Hypnic Headache Syndromes) OR (Alarm Clock Headache) OR (Alarm Clock Headaches) OR (Clock Headache, Alarm) OR (Clock Headaches, Alarm) OR (Headache, Alarm Clock) OR (Headaches, Alarm Clock)<br>#5 MeSH descriptor: [Low-Level Light Therapy] explode all trees<br>#6(Light Therapies, Low-Level) OR (Light Therapy, Low-Level) OR (Low Level Light Therapy) OR (Low-Level Light Therapies) OR (Therapies, Low-Level Light) OR (Therapy, Low-Level Light) OR (Photobiomodulation Therapy) OR (Photobiomodulation Therapies) OR (Therapies, Photobiomodulation) OR (Therapy, Photobiomodulation) OR (LLLT) OR (Laser Therapy, Low-Level) OR (Laser Therapies, Low-Level) OR (Laser Therapy, Low Level) OR (Low-Level Laser Therapies) OR (Laser Irradiation, Low-Power) OR (Irradiation, Low-Power Laser) OR (Laser Irradiation, Low Power) OR (Low-Power Laser Therapy) OR (Low Power Laser Therapy) OR (Laser Therapy, Low-Power) OR (Laser Therapies, Low-Power)

	OR (Laser Therapy, Low Power) OR (Low-Power Laser Therapies) OR (Low-Level Laser Therapy) OR (Low Level Laser Therapy) OR (Low-Power Laser Irradiation) OR (Low Power Laser Irradiation) OR (Laser Biostimulation) OR (Laser Phototherapy) OR (Phototherapy, Laser)<br>#7#1 OR #2 OR #3 OR #4<br>#8#5 OR #6<br>#9#7 AND #8 (In Trials) (110)
**Literatura Latino-Americana em Ciências da Saúde e do Caribe — LILACS (via Biblioteca Virtual em Saúde—BVS)**	#1 MH: Cefaleia OR Headache OR Cefalea OR C23.888.592.612.441<br>#2MH: (Terapia com Luz de Baixa Intensidade) OR (Low-Level Light Therapy) OR(Terapia por Luz de Baja Intensidad) OR E02.594.540 OR E02.774.500<br>#4 #2 OR #3<br>#5 #1 AND #4 AND (db:("LILACS")) (2)
**PEDro (PhysiotherapyEvidenceDatabase)**	Abstract & Title: Headache<br>Therapy: electrotherapies, heat, cold<br>Problem: pain<br>Body part: head or neck<br>Method: clinicaltrial (81)
**PsycInfo (via APA)**	#1{Headache}<br>#2 (Low-Level Light Therapy) OR (Light Therapies, Low-Level) OR (Light Therapy, Low-Level) OR (Low Level Light Therapy) OR (Low-Level Light Therapies) OR (Therapies, Low-Level Light) OR (Therapy, Low-Level Light) OR (Photobiomodulation Therapy) OR (Photobiomodulation Therapies) OR (Therapies, Photobiomodulation) OR (Therapy, Photobiomodulation) OR (LLLT) OR (Laser Therapy, Low-Level) OR (Laser Therapies, Low-Level) OR (Laser Therapy, Low Level) OR (Low-Level Laser Therapies) OR (Laser Irradiation, Low-Power) OR (Irradiation, Low-Power Laser) OR (Laser Irradiation, Low Power) OR (Low-Power Laser Therapy) OR (Low Power Laser Therapy) OR (Laser Therapy, Low-Power) OR (Laser Therapies, Low-Power) OR (Laser Therapy, Low Power) OR (Low-Power Laser Therapies) OR (Low-Level Laser Therapy) OR (Low Level Laser Therapy) OR (Low-Power Laser Irradiation) OR (Low Power Laser Irradiation) OR (Laser Biostimulation) OR (Laser Phototherapy) OR (Phototherapy, Laser)<br>#3 #1 AND #2 (292)

**Clinicaltrials.gov**	Headache AND (Photobiomodulation Therapy) (1)
**WHO/ICTRP**	Headache AND (Photobiomodulation Therapy) (0)
**Opengrey**	Headache AND (Photobiomodulation Therapy) (0)
